# CeO_2_NP priming enhances the seed vigor of alfalfa (*Medicago sativa*) under salt stress

**DOI:** 10.3389/fpls.2023.1264698

**Published:** 2024-01-09

**Authors:** Jinzhu Gao, Yanzhi Liu, Donghao Zhao, Yanzhi Ding, Le Gao, Xihao Su, Kexiao Song, Xueqing He

**Affiliations:** College of Grassland Agriculture, Northwest A&F University, Yangling, Shaanxi, China

**Keywords:** nanoparticle priming, *Medicago sativa*, salt stress, CeO_2_NPs, plant metabolism

## Abstract

Soil salinization is a common environmental problem that seriously threatens crop yield and food security, especially through its impact on seed germination. Nanoparticle priming, an emerging seed treatment method, is receiving increasing attention in improving crop yield and stress resistance. This study used alfalfa seeds as materials to explore the potential benefits of cerium oxide nanoparticle (CeO_2_NP) priming to promote seed germination and improve salt tolerance. CeO_2_NPs at concentrations up to 500 mg/L were able to significantly alleviate salt stress in alfalfa seeds (200 mM), with 50 mg/L of CeO_2_NP having the best effect, significantly (*P<* 0.05) increasing germination potential (from 4.0% to 51.3%), germination rate (from 10.0% to 62.7%), root length (from 8.3 cm to 23.1 cm), and seedling length (from 9.8 cm to 13.7 cm). Priming treatment significantly (*P<* 0.05) increased seed water absorption by removing seed hardness and also reducing abscisic acid and jasmonic acid contents to relieve seed dormancy. CeO_2_NP priming increased α-amylase activity and osmoregulatory substance level, decreased reactive oxygen species and malonaldehyde contents and relative conductivity, and increased catalase enzyme activity. Seed priming regulated carotenoid, zeatin, and plant hormone signal transduction pathways, among other metabolic pathways, while CeO_2_NP priming additionally promoted the enrichment of α-linolenic acid and diterpenoid hormone metabolic pathways under salt stress. In addition, CeO_2_NPs enhanced α-amylase activity (by 6.55%) *in vitro*. The optimal tested concentration (50 mg/L) of CeO_2_NPs was able to improve the seed vigor, enhance the activity of α-amylase, regulate the osmotic level and endogenous hormone levels, and improve the salt tolerance of alfalfa seeds. This study demonstrates the efficacy of a simple seed treatment strategy that can improve crop stress resistance, which is of great importance for reducing agricultural costs and promoting sustainable agricultural development.

## 1 Introduction

Soil salinization is a worldwide soil and environmental problem caused by natural and human activities. According to incomplete statistics, the global area of alkali soil has reached approximately 1 billion hectares, accounting for 7% of the total land area; the area of secondary alkali soil has reached 77 million hectares, of which 58% is concentrated in irrigated areas (Yang et al., 2022), and this percentage is growing. China’s climate conditions, ecological environment, and geographical terrain are complex and diverse, but soil salinization is a common problem that is distributed across almost all regions of the country, severely restricting agricultural development (Barradas et al., 2015; Chen et al., 2019). As a key abiotic stress that affects one-quarter to one-third of the crop productivity of agricultural soils, salt stress has severe negative effects on seed germination, crop growth, and productivity (Munns, 2002; Yang and Guo, 2018). As the world population increases, more food is needed to meet the rising demand, and the use of saline land to compensate for the resulting food shortage is becoming more urgent (Qin and Huang, 2020). Alfalfa is a perennial leguminous forage with strong adaptability and high nutritional value. Salt stress is the main abiotic stress factor affecting the yield and nutritional quality of alfalfa (Rajendran et al., 2009). Therefore, it is critical to improve the salt tolerance of alfalfa in order to make better use of saline land, meet the needs of animal husbandry, and ensure food security. To improve germplasm in order to enhance crop stress resistance and yield and further improve the utilization efficiency of saline–alkali land, agricultural scientists have taken different measures, e.g., breeding new varieties (Rumanti et al., 2018) and developing seed presowing treatments (Liu et al., 2021). One of the most frequently utilized measures is seed priming (Ibrahim, 2016), which is a successful approach to induce stress tolerance in plants (Khan et al., 2023). Seed priming is defined as pre-exposure of seeds to an eliciting factor known to “prepare” the plants for future stress, thus resulting in greater survival under adverse environmental conditions (Tanou et al., 2012). The primed state of a plant refers to the physiological state in which plants can activate stress tolerance mechanisms faster or better or both while improving seed vigor to synchronize seed germination under unfavorable growing conditions (Marthandan et al., 2020).

With the advent of new agricultural technology, nanotechnology has begun to play an undeniable role. For agriculture applications, many nanomaterials have been developed, including innovative soil and water remediation solutions, nanofertilizers, and nanopesticides that aim to reduce fertilizer and pesticide application while enhancing food yield and quality (Do et al., 2021). Compared with foliar and basal applications, nanopriming is a more cost-effective and environmentally friendly strategy to increase seed quality, promote growth, and increase yields (Amritha et al., 2021; Khan et al., 2021). This type of approach could not only reduce the release of engineered nanomaterials in the environment (as less overall material is used) but also result in decreased worker exposure to these materials (Zhao et al., 2022). At present, rare earth elements (REEs), including REE nanomaterials, have become indispensable in many sectors (Migaszewski and Galuszka, 2015). Cerium (Ce) is the most abundant REE in the continental crust of the earth (Pourkhorsandi et al., 2021), while cerium oxide nanoparticles (CeO_2_NPs) have been widely used for seed priming, such as in the use of polyacrylic-acid-coated nanoceria, which has improved rapeseed (9.2 nm, −38.7 mV) (Khan et al., 2021; Khan et al., 2022), cotton (6.05/8.04 nm, −15.30 mV) (Liu et al., 2021), and *Arabidopsis* (10.3 nm, −16.9 mV, 50 mg/L) (Wu et al., 2018) salt tolerance. Uncoated CeO_2_NPs (620.7 nm, −11.6 mV, 500 mg/L) were reported to significantly alleviate DNA damage in NaCl-treated rice (Wang et al., 2019). However, up to the present, there have been few reports on seed priming using CeO_2_NPs to improve salt tolerance in alfalfa, and there is relatively little research comparing and analyzing the effects of nanopriming with hydropriming. Accordingly, it is important to explore possible methods to improve salt tolerance in plant seeds using CeO_2_NPs as the priming material.

Seed germination is the first step of establishing a new plant. Under salt stress, seed germination is affected by osmotic stress, ion-specific effects, and oxidative stress, as evidenced by a lower germination rate and/or longer germination time (Munns, 2002). Salinity can affect seed germination by reducing the amount of seed germination stimulants like gibberellic acids GAs, increasing the amount of abscisic acid (ABA), and altering seed membrane permeability and water potential (Lee and Luan, 2012). The transcriptional activation of hydrolytic enzymes such as α-amylase is the hallmark of seed germination (Damaris et al., 2019). The α-amylase enzyme is released into the endosperm to start breaking down the stored starch into soluble sugars, which supply energy and nutrients to the developing embryo and radicle (Khan et al., 2023). Deprivation of water accompanied by a reduction in α-amylase activity with increased NaCl content may be the leading cause of germination delays (Kaneko et al., 2002). Moreover, decreasing soluble sugar content alters the osmotic potential of developing cells, thus lowering water intake (El-Hendawy et al., 2019). As such, salt stress exerts osmotic and ionic impacts that produce excessive reactive oxygen species (ROS) and result in oxidative damage, thus damaging proteins, lipids, nucleic acids, and the cellular structure (Hanin et al., 2016). A large number of ROS-detoxifying proteins are present in cells to mitigate oxidative stress (Miller et al., 2010). We attempt to explore the mechanisms by which nanopriming enhances salt tolerance from these perspectives.

As a new seed priming technology, nanopriming has the advantages of short exposure time, breaking seed dormancy, and improving seed vigor. Based on this, we applied CeO_2_NPs to study their effect on the stress resistance of alfalfa seeds. The present study was undertaken to answer the following questions. 1) Does CeO_2_NP priming have beneficial effects on the salt tolerance of alfalfa seeds, and if so, what are those effects? 2) Does CeO_2_NP priming improve salt tolerance and promote germination better than hydropriming? 3) What is the mechanism behind the difference in efficacy of CeO_2_NP priming and hydropriming? This study explored and analyzed seed coat structure, elemental composition, physiological and biochemical responses, and hormone metabolism in order to address these questions.

## 2 Materials and methods

### 2.1 Source and characterization of nanoparticles and in-vitro experiments on nanocatalysis of α-amylase

Analytical grade CeO_2_NPs (99.5%, 20–50 nm, CAS 1306-38-3) were purchased from Aladdin Reagent Company (www.aladdin-e.com, Shanghai, China). The seeds of the alfalfa variety ‘Huanghou’ were purchased from Jiangsu Qianhuabaimei Seed Industry Co., Ltd. (Suqian, China) in May 2022.

To determine the shape and size of CeO_2_NPs, the CeO_2_NPs were dissolved in distilled water, and a drop of CeO_2_NP sample was placed on a Cu grid and dried under vacuum for analysis. The prepared samples were observed using a transmission electron microscope (TEM) (Hitachi High-Technologies Corporation, HT7800, Tokyo, Japan) at an accelerating voltage of 120 kV. To characterize the size and zeta potential of CeO_2_NPs, the samples were made into a suspension and measured with a nanometer laser particle sizer (ZEN3600; Malvern Instruments Limited, Malvern, UK). The crystal properties of CeO_2_NPs were determined by X-ray diffraction (XRD) analysis. The sample was also subjected to an X-ray diffractometer (Model D8 Advance A25; Bruker, Bremen, Germany) at 40 kV and 40 mA with Cu Kα1 radiation. The scan 2*θ* range was 10–80°.

To investigate whether CeO_2_NPs have nanocatalytic activity against α-amylase, *in-vitro* experiments were conducted. The *in-vitro* experiment was adjusted according to the α-amylase activity measurement method. In short, 2 mL of 0, 50, 100, and 500 mg/L CeO_2_NP solutions were mixed with 8 mL of α-amylase solution (3.7 U/mL) to form a mixed solution of 0, 10, 50, and 100 mg/L CeO_2_NPs and incubated at 37°C for 2 h, and then, Kishorekumar’s (2007) method was used to determine the enzymatic activity of the mixed solution. In short, after incubation, the different amylase solutions were mixed with starch solutions; after sufficient reaction time, the absorbance value was measured using a spectrophotometer, and the enzyme activity was calculated using a standard curve for the glucose system. Each treatment was repeated three times.

### 2.2 Selection of salt concentration for alfalfa germination

Salt (NaCl) solutions with different concentrations (0, 50, 100, 150, 200, and 250 mM) were established to conduct germination experiments to characterize the effects of salt concentration. Referring to the processing method of Song et al. (2021), all seeds used for the germination experiment were disinfected with 75% alcohol for 30 s and washed five times with sterile water. For each treatment, seeds were germinated on double-layer filter paper in Petri dishes with 5 mL of treatment solution. Then, the seeds were put in a germination chamber maintained at 25°C ± 2°C, 85% relative humidity, and a 16/8-h photoperiod (light/dark) with 10,000 lx irradiance for 10 days; water was replenished daily to keep the filter paper and seeds wet. This was the setup used to determine the germination potential (GP, 4 days), germination rate (GR, 10 days), and root length (RL) and shoot length (SL) at the germination stage.

Univariate quadratic regression analyses were performed on the relative values of various test indicators and salt solution concentrations of alfalfa based on the following equation:


$$\text{Equation}:y=ax^{2}+bx+c.$$

Here, *a*, *b*, and *c* are equation coefficients (Li et al., 2019).

The salt concentration at 50% lethality (*S*_50_) was calculated based on this regression equation, and the mean *S*_50_ values of each indicator were calculated to obtain the screening concentration.

### 2.3 Preparation of the nanopriming solution and determining the priming concentration

CeO_2_NP suspension solutions were used as nanopriming solutions. The particles were dispersed in deionized water by ultrasonic vibration (100 W, 40 kHz) with an ultrasonic cleaner (Ningbo Scientz Biotechnology Co., Ltd., Ningbo, China) for 30 min to prepare the nanopriming solutions of corresponding concentrations.

The CeO_2_NPs were dispersed in deionized water to prepare nanopriming solutions with different concentrations (0, 10, 50, 100, 200, 500 mg/L). Six batches of seeds (500 seeds for each) with uniform size and full granules were placed into PE tubes with plugs, to which 20 mL of nanopriming solutions of various concentrations were added. After the seeds were soaked for 24 h (in the dark, 10°C), seeds were dried back at room temperature for 48 h and stored for later use. Control (CK, non-priming) and primed seeds were germinated under salt treatment (using the concentration identified in the previous section). The seed germination conditions were the same as those described in the previous section (2.2).

Referring to the method of Li et al. (2019), evaluations were conducted of the salt tolerance of each priming concentration by determining its salt tolerance membership function value.


Equation:Index of salt tolerance=NaCl treatment value/control value.

$$\text{Equation}:X_{i}=\left(X-X_{min}\right)/\left(X_{max}-X_{min}\right)*100\%.$$

Here, *X_i_* is the membership function value of the salt tolerance index for treatment concentration *i*, *X* is the actual measured value of the salt tolerance index, and *X*_max_ and *X*_min_ are the maximum and minimum values of the salt tolerance index for each concentration treatment, respectively. First, the membership function values were calculated for each indicator and summed, and then, the average membership function values were calculated to evaluate salt tolerance under different concentration treatments. The average range was between 0 and 1, and the larger the average, the higher the salt tolerance. The optimal priming concentration was used for the subsequent experiments.

### 2.4 Ultrastructural observation of primed seeds

In order to better observe changes in the epidermal structure of seeds after priming treatment, verify whether these changes provide conditions for water absorption and nanomaterial infiltration, and further verify Ce penetrates the seed coat, we conducted ultrastructural observations of the seed coat and cross-section. Seeds were collected from different treatments [non-primed (CK), hydropriming (HP), CeO_2_NP priming (Ce)], and their epidermises were observed by scanning electron microscopy (SEM; JSM 5600 LV; JEOL, Tokyo, Japan). Additionally, an energy-dispersive X-ray spectrometer (EDX; KEVE2; Hillsboro, USA) was used for the point analysis of epidermal elements. CeO_2_NP-primed seeds were cut along the cotyledon–radicle axis, and the longitudinal section was observed under SEM, and elemental line scanning was conducted (on the cotyledon–radicle sample) to analyze the distribution of Ce.

### 2.5 Seed water absorption and α-amylase activity

Water absorption is an important factor affecting seed germination, and water can activate starch hydrolases to provide material reserves for germination. Therefore, seed water absorption and α-amylase activity were measured. Approximately 0.05 g of alfalfa seeds were tested for their seed water absorption rate for 24 h in a germination environment, with three replicates. Seeds were weighed at 0, 3, 6, 9, 16, and 24 h, respectively, and the seed water absorption rates were calculated for different treatments (i.e., CK, HP, and Ce). The activity of α-amylase was measured by the 3,5-dinitrosalicylic acid method (Kishorekumar et al., 2007). Specifically, samples (0.1 g) of alfalfa seeds that had germinated for 24 h under different treatments were homogenized and brought to a constant volume of 1.5 mL. Samples were extracted at room temperature for 15 min and then centrifuged at 6,000×*g* for 10 min, and the resulting supernatant was collected as the enzyme extract. Then, a spectrophotometer was used to measure the absorbance value, and thus, it was possible to calculate enzyme activity based on a standard curve for the glucose system.

### 2.6 Relative electrical conductivity and malondialdehyde content

Relative conductivity (REC) and malondialdehyde (MDA) content can indicate the degree of cellular damage. REC was measured according to the method described by Zou (2003). In detail, 0.1 g of germinated seeds (after 24 h) was washed three to five times with deionized water, and after the water was wiped away, 5 mL of deionized water was added. The electrical conductivity of the leachate when the seed was first added was measured with a conductivity meter (DDSJ – 318; Rex Electric Chemical, Jiashan, China) as the initial electrical conductivity (A1). After samples were placed under vacuum with a vacuum pump for 10 min, the conductivity (A2) of leachate after standing for 30 min was measured. After boiling the leachate in a 100°C water bath for 15 min and then cooling it to 25°C, the conductivity of the leachate was measured again as the absolute conductivity (A3). The formula for REC is as follows: REC (%) = (A2 − A1)/(A3 − A1) * 100. Malonaldehyde (MDA) content was determined following the approach of Heath and Packer (1968).

### 2.7 Hydrogen peroxide and superoxide anion content

ROS can act as signaling molecules and also serve as an indicator of stress level. Seeds that had germinated for 24 h (0.2 g, *n* = 3) were placed into 3.0 mL of PBS (50 mM, pH 7.8) on ice and ground into a homogenate that was then centrifugated (12,000 r/min, 4°C) for 5 min, and the resulting supernatant was used as the sample extract solution for the determination of H_2_O_2_ and O_2_^·−^ contents. The determination of H_2_O_2_ content was carried out using the method of Ferguson et al. (1983). The content of O_2_^·−^ was determined by the hydroxylamine oxidation method (Ke et al., 2002).

### 2.8 Soluble proteins, amino acids, and soluble sugar content

Soluble proteins, amino acids, and soluble sugar content can be used as both osmotic regulators to alleviate osmotic stress and as nutrients for seed germination. Therefore, their contents should be measured when evaluating stress responses. The concentration of amino acid content in germinated seeds was measured by the ninhydrin method (Zou, 2003) and quantified using leucine as the standard. The supernatants containing the amino acids from germinated seeds were assayed for soluble protein content by the Bradford method (Bradford, 1976). The soluble sugar content of the germinated seeds was measured by the anthrone colorimetric method (Zou, 2003) using glucose as the standard.

### 2.9 Determination of superoxide dismutase, peroxidase, and catalase enzyme activity

Antioxidant enzymes play a crucial role in clearing harmful oxidative substances from cells. Superoxide dismutase (SOD) activity was calculated as the units of enzyme required for 50% inhibition of nitroblue tetrazolium per gram of fresh seeds per minute according to the approach of Beauchamp and Fridovich (1971). Catalase (CAT) activity was measured according to the approach of Aebi (1984). The reduction in absorbance owing to H_2_O_2_ degradation was measured at 240 nm (*A*_240_). A 0.1 decrease in *A*_240_ within 1 min was defined as one unit of CAT enzyme activity. Peroxidase (POD) activity was analyzed following the method of Hemeda and Klein (1990). An increase of 0.01 in *A*_470_ per minute was defined as one unit of POD enzyme activity.

### 2.10 Determination of hormone content and metabolic differences in seeds

The role of plant hormones as signaling molecules during seed germination cannot be ignored, and metabolic analysis can help to elucidate the underlying causes of germination differences. Alfa seeds (0.1 g) germinated under different treatments for 24 h were used as sample material for the measurement of hormone metabolism levels. Fresh seeds were harvested, immediately frozen in liquid nitrogen, ground into powder (30 Hz, 1 min), and stored at −80°C until subsequent analysis. Fifty-milligram samples of seeds were weighed into 2-mL plastic microtubes, frozen in liquid nitrogen, and dissolved in 1 mL of methanol/water/formic acid (15:4:1, *V*:*V*:*V*). Ten microliters of an internal standard mixed solution (100 ng/mL) was added into the extract as internal standards (IS) for quantitation. The mixture was vortexed for 10 min and then centrifuged for 5 min (12,000 r/min, 4°C), and the resulting supernatant was transferred to clean plastic microtubes, evaporated to dryness and dissolved in 100 μL of 80% methanol (*V*/*V*), and filtered through a 0.22-μM membrane filter for further LC-MS/MS analysis.

### 2.11 Statistical analysis

A one-way ANOVA was performed to determine the effects on the means of treatment samples across three replications. Data were analyzed using SPSS 26.0 (IBM Corp, Armonk, NY, USA). The significance levels of differences among the means of all the measured traits were determined by Duncan’s multiple range test at a 5% level. Values of *P* smaller than or equal to 0.05 were considered statistically significant.

## 3 Results

### 3.1 Characterization of CeO_2_NPs

**Figure 1:**
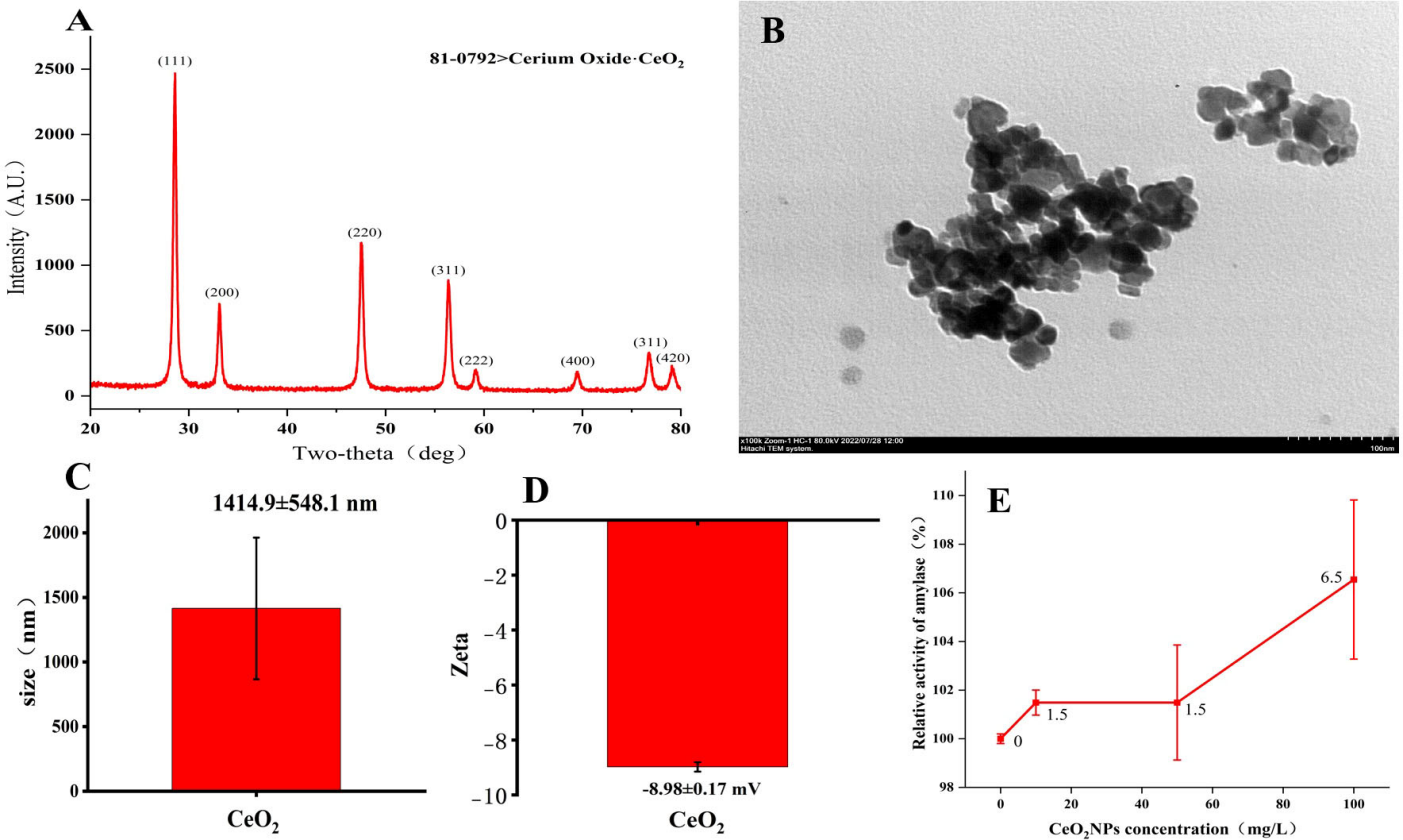
Characterization results of cerium oxide nanoparticles (CeO_2_NPs). X-ray diffraction results **(A)**; transmission electron microscopy image **(B)**; mean size **(C)**; mean Zeta **(D)**; relative activity of amylase as a function of CeO_2_NP concentration based on *in-vitro* experiments **(E)**.

**Figure 1A** shows the XRD pattern of cerium oxide (CeO_2_) nanoparticles, with card ID 81-0792. The TEM image of typical CeO_2_NPs in **Figure 1B** shows a loose cluster composed of uniformly sized nanoparticles. The hydrodynamic fluid size of CeO_2_NPs is 1,414.9 ± 548.1 nm (**Figure 1C**), while the zeta potential analysis results are −8.98 ± 0.17 mV (**Figure 1D**). In the *in-vitro* experiments, as the concentration of CeO_2_NPs increased, the amylase activity also increased, and the 100-mg/L treatment achieved the maximum increase in enzyme activity, an increase of 6.55% (**Figure 1E**).

### 3.2 Effects of different concentrations of salt stress on the germination of alfalfa seeds

**Figure 2:**
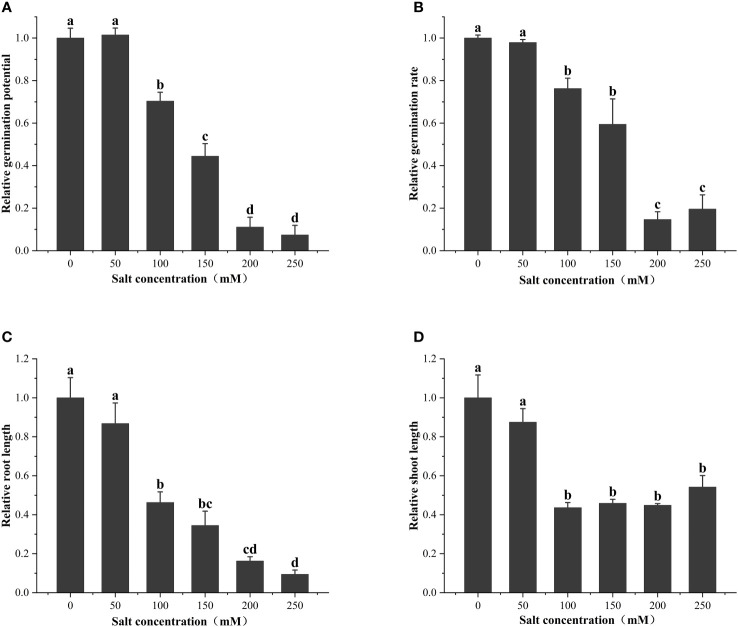
Effect of salt treatments of different concentrations on **(A)** relative germination potential, **(B)** relative germination rate, **(C)** relative root length, and **(D)** relative shoot length at the seed germination stage. These data are the average of three replications (*n* = 3). Different letter labels on the columns in the figure indicate significant differences between different treatments (*P<* 0.05).

As shown in **Figure 2**, the seed vigor of alfalfa seeds decreased with increases in salt concentration. Specifically, the salt concentration of 100 mM significantly (*P<* 0.05) reduced the germination potential (29.63%), germination rate (23.78%), root length (53.69%), and seedling length (56.35%) of alfalfa during the germination period. *S*_50_ was calculated based on the salt damage membership function and estimated to be 202.1 mM (**Table 1**); thus, subsequent experiments were based on the salt concentration of 200 mM.

**Table 1:** Salt damage membership function index and concentration of salt at 50% lethality (*S*_50_) of alfalfa.

Equation coefficient	Value				Average
	Relative germination potential	Relative germination rate	Relative root length	Relative shoot length	
*a*	−2.487 * 10^−6^	−4.1 * 10^−6^	9.136 * 10^−6^	−3.7 * 10^−6^	
*b*	−0.004	−0.003	−0.004	−0.001	
*c*	1.08	1.056	1.021	1.01	
*S*_50_ (mM)	138.5	154.6	243.3	271.9	**202.1**

The bold value means the average value of S50, which is the concentration reference for selecting salt stress in the following text.

### 3.3 The effect of CeO_2_NP priming on seed vigor under salt stress

**Figure 3:**
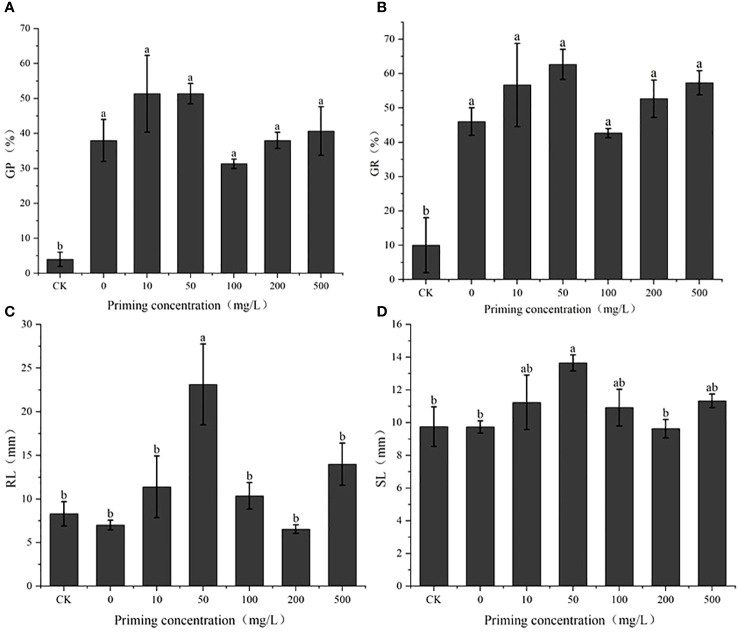
The effect of cerium oxide nanoparticle (CeO_2_NP) priming on seed vigor under salt stress compared with control treatment (CK). Germination potential (GP) **(A)**; germination rate (GR) **(B)**; root length (RL) **(C)**; shoot length (SL) **(D)**. Different letter labels on the columns in the figure indicate significant differences between different treatments (*P*< 0.05).

Salt concentration screening identified the *S*_50_ value as 200 mM (**Figure 2**; **Table 1**). On this basis, the effects of different concentrations of CeO_2_NPs on the seed vigor of alfalfa under salt stress were studied (**Figure 3**). Priming treatment was able to improve seed germination potential and germination rate, as well as the root and seedling length during seedling germination under salt stress. Different concentrations of CeO_2_NPs had a significant (*P<* 0.05) promoting effect on alfalfa seeds under salt stress. The optimal effect was achieved at a concentration of 50 mg/L (**Table 2**), with the germination potential increasing from 4.0% to 51.3% (**Figure 3A**) and the germination rate increasing from 10.0% to 62.7% (**Figure 3B**), significantly (*P<* 0.05) improving the growth of alfalfa seedlings during the germination period, increasing the root length from 8.3 cm to 23.1 cm (**Figure 3C**), and promoting the growth of seedlings from 9.8 cm to 13.7 cm (**Figure 3D**).

**Table 2:** Priming concentration screening results.

Priming concentration	STMF-GP	STMF-GR	STMF-RL	STMF-SL	STMF-average
CK	0.00	0.00	0.11	0.03	0.04
0	0.72	0.68	0.03	0.03	0.37
10	1.00	0.89	0.29	0.40	0.65
50	1.00	1.00	1.00	1.00	**1.00**
100	0.58	0.62	0.23	0.32	0.44
200	0.72	0.81	0.00	0.00	0.38
500	0.77	0.90	0.45	0.42	0.64

STMF, salt tolerance membership function; GP, germination potential; GR, germination rate; RL, root length; SL, shoot length.

The bold values represent the maximum values in the average salt tolerance membership function, which serve as the basis for selecting the 50 mg/L treatment for the next experiment.

### 3.4 Ultrastructural observation, element distribution analysis, and water absorption of primed seeds

**Figure 4:**
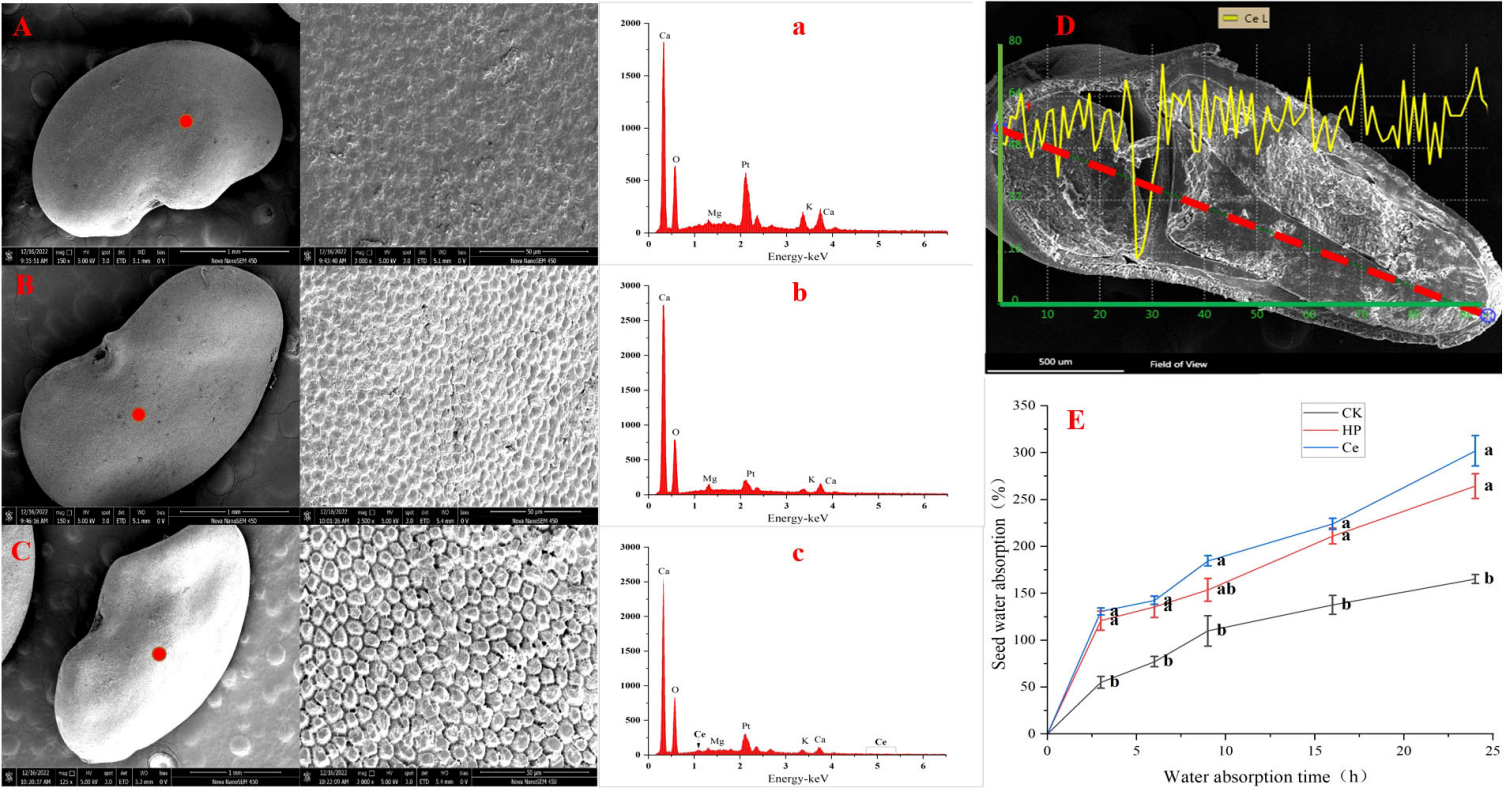
Characterization of seeds under different treatments. Observation of the seed epidermis: control (CK) **(A)**, hydropriming (HP) **(B)**, cerium oxide nanoparticle priming (Ce) **(C)**; energy dispersive X-ray spectrometry (EDX) analysis pattern of the seed epidermis: CK (a), HP (b), Ce (c); seed profile analysis **(D)**; water absorption curve **(E)**, respectively.

Through observing the alfalfa seeds under different treatments by SEM, it was found that the priming treatment had a significant impact on the seed coat structure (**Figure 4**). The non-primed seeds were plump and smooth, with tightly arranged epidermal cells faintly visible, and the surface was covered with a waxy layer (**Figure 4A**). Under hydropriming, rough epidermis and cracks between epidermal cells appeared (**Figure 4B**). Under CeO_2_NP priming, the epidermis of the seeds was rough, with the wax layer fully consumed; additionally, the epidermal cells were loose, and the gaps between the epidermal cells were obvious (**Figure 4C**). Thus, it could be seen that priming treatment promoted the erosion of the seed coat wax and loosened the epidermal cells. Accordingly, the effect of CeO_2_NP priming was visibly better than that of hydropriming. Using EDX point scanning analysis, it was found that cerium deposition was present in the seed epidermis of seeds that had been primed by CeO_2_NPs, with concentrations of 0.03% (atomic) and 0.3% (weight), while there was no cerium deposition in the hydroprimed and non-primed seed epidermis (**Figures 4A–C**; **Table 3**). Through EDX line-scanning analysis on the seed profile, it was determined that CeO_2_NPs fully entered the radicle and cotyledon tissues of alfalfa seeds and were evenly distributed (**Figure 4D**). Compared with the non-primed control treatment, the seed water absorption curve (**Figure 4E**) indicated that priming treatments (including hydropriming and CeO_2_NP priming) can significantly improve the water absorption of alfalfa seeds. In addition, the water absorption effect of seeds primed with CeO_2_NPs was better than that of hydroprimed seeds.

**Table 3:** Analysis and comparison of elements in the seed epidermis of alfalfa under different control (CK), hydropriming (HP), and cerium oxide nanoparticle priming (Ce) treatments.

Treatment	Contents							
		C	N	O	K	Ca	Mg	Ce
CK	Atom%	17.36	51.77	27.86	0.63	0.87	0.6	—
	Weight%	12.8	44.49	27.35	1.51	2.15	0.89	—
HP	Atom%	13.11	50.67	34.44	0.26	0.59	0.68	—
	Weight%	10.37	46.75	36.3	0.67	1.55	1.09	—
Ce	Atom%	11.73	47.71	38.67	0.26	0.51	0.48	0.03
	Weight%	8.84	41.93	38.82	0.64	1.2	0.74	0.3

### 3.5 α-Amylase activity and germination substance levels

**Figure 5:**
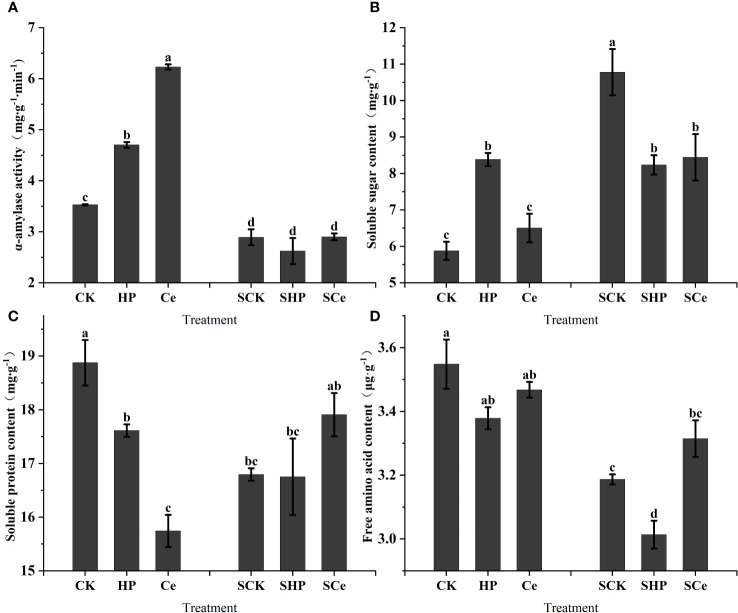
Effects of different treatments on key hydrolase and germination substance levels in germinated seeds. α-Amylase activity **(A)**; soluble sugar content **(B)**; soluble protein content **(C)**; free amino acid content **(D)**. Different letter labels on the columns in the figure indicate significant differences between different treatments (*P*< 0.05). SCK, SHP, and SCe refer to seeds germinated under control, hydropriming, and cerium oxide nanoparticle priming treatments, respectively, under salt stress, while CK, HP, and Ce refer to seeds germinated under control, hydropriming, and cerium oxide nanoparticle priming treatments, respectively, without salt stress.

As shown in **Figure 5A**, hydropriming and CeO_2_NP priming treatments significantly (*P<* 0.05) increased the α-amylase activity of alfalfa seeds, and the promotion effect caused by CeO_2_NP priming was better than that of hydropriming. Additionally, salt stress significantly (*P<* 0.05) decreased α-amylase activity. The hydropriming treatment significantly (*P<* 0.05) increased the soluble sugar content of germinating alfalfa seeds, but both hydropriming and CeO_2_NP priming treatment significantly (*P<* 0.05) decreased the soluble sugar content compared with the control under salt stress (**Figure 5B**). Overall, salt stress increased the soluble protein content of germinated seeds. **Figure 5C** shows that the priming treatment significantly reduced the soluble protein content of germinated seeds, and the CeO_2_NP priming treatment had the lowest content. However, CeO_2_NP priming relatively increased the soluble protein content under salt stress. Compared with the control treatment, hydropriming reduced free amino acid content, and the free amino acid content under CeO_2_NP priming was higher than that under hydropriming; salt stress reduced free amino acid content in general (**Figure 5D**).

### 3.6 Effects of different treatments on membrane damage of germinated seeds

**Figure 6:**
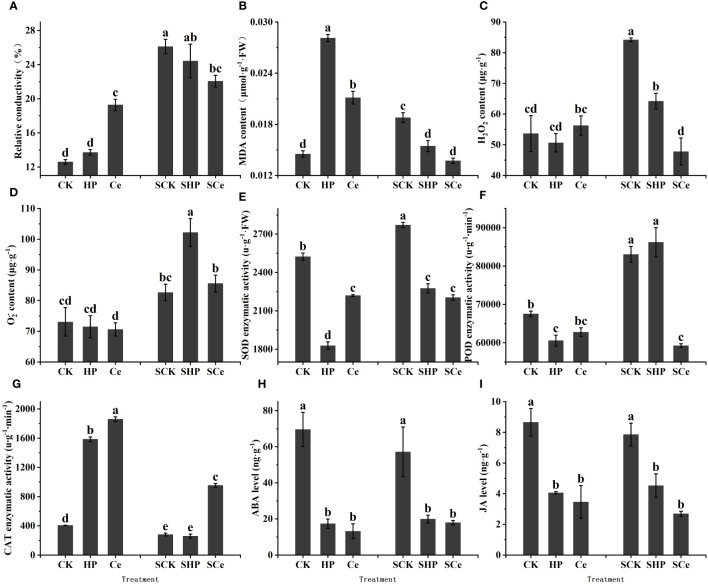
Effects of different treatments on physiological indicators and contents of key hormones of germinated seeds. Relative conductivity **(A)**; malonaldehyde (MDA) content **(B)**; reactive oxygen species (ROS) content **(C, D)**; superoxide dismutase (SOD) activity **(E)**; peroxidase (POD) activity **(F)**; catalase (CAT) activity **(G)**; abscisic acid (ABA) content **(H)**; jasmonic acid (JA) content **(I)**, respectively. Different letter labels on the columns in the figure indicate significant differences between different treatments (*P*< 0.05).

Compared with the control and hydropriming treatments, CeO_2_NP priming significantly (*P<* 0.05) increased the relative conductivity of germinated seeds under control conditions and significantly (*P<* 0.05) reduced conductivity under salt stress. Overall, salt stress increased the conductivity of germinated seeds (**Figure 6A**). Hydropriming and CeO_2_NP priming significantly (*P<* 0.05) increased the MDA content of germinated seeds, while CeO_2_NP-primed seeds had lower MDA content. Both hydropriming and CeO_2_NP priming significantly (*P<* 0.05) reduced MDA content under salt stress (**Figure 6B**).

### 3.7 Effects of different treatments on ROS content of germinated seeds

As shown in **Figures 6C, D**, under control conditions, the priming treatment had no significant effect on the ROS content of germinated seeds. However, under salt stress, both hydropriming and CeO_2_NP priming reduced the H_2_O_2_ content of the seeds, while hydropriming increased the O_2_^.−^ content of the seeds. CeO_2_NP priming significantly (*P<* 0.05) reduced H_2_O_2_ and O_2_^.−^ contents compared with hydropriming.

### 3.8 Effects of different treatments on antioxidant enzyme activity of germinated seeds

Hydropriming and CeO_2_NP priming significantly (*P<* 0.05) reduced SOD enzyme activity, with the activity of hydropriming promoting the lowest level. Under salt stress, the SOD enzyme activity of primed seeds significantly decreased, with CeO_2_NP priming promoting the lowest level (**Figure 6E**). Priming reduced the POD enzyme activity of germinated seeds, while only CeO_2_NP priming significantly (*P<* 0.05) reduced the POD enzyme activity under salt stress (**Figure 6F**). The two priming treatments (HP and Ce) significantly (*P<* 0.05) increased the CAT enzyme activity of germinated seeds, while only CeO_2_NP priming significantly (*P<* 0.05) increased the CAT enzyme activity under salt stress (**Figure 6G**).

### 3.9 Effects of different treatments on abscisic acid and jasmonic acid levels of germinated seeds

ABA is mainly a hormone that promotes seed dormancy and inhibits germination (Guillaume et al., 2017), while jasmonic acid (JA) hormones are also important hormones that promote seed dormancy and may interact with ABA. Both under control and salt stress conditions, priming treatments significantly (*P<* 0.05) reduced the ABA content of germinated alfalfa seeds, and the ABA levels under hydropriming and CeO_2_NP priming were similar (**Figure 6H**). Priming significantly (*P<* 0.05) reduced the JA content of alfalfa seeds under control and salt stress, and CeO_2_NP priming promoted lower JA content compared with hydropriming (**Figure 6I**).

### 3.10 Statistical analysis of metabolites in germinated seeds under different treatments

**Figure 7:**
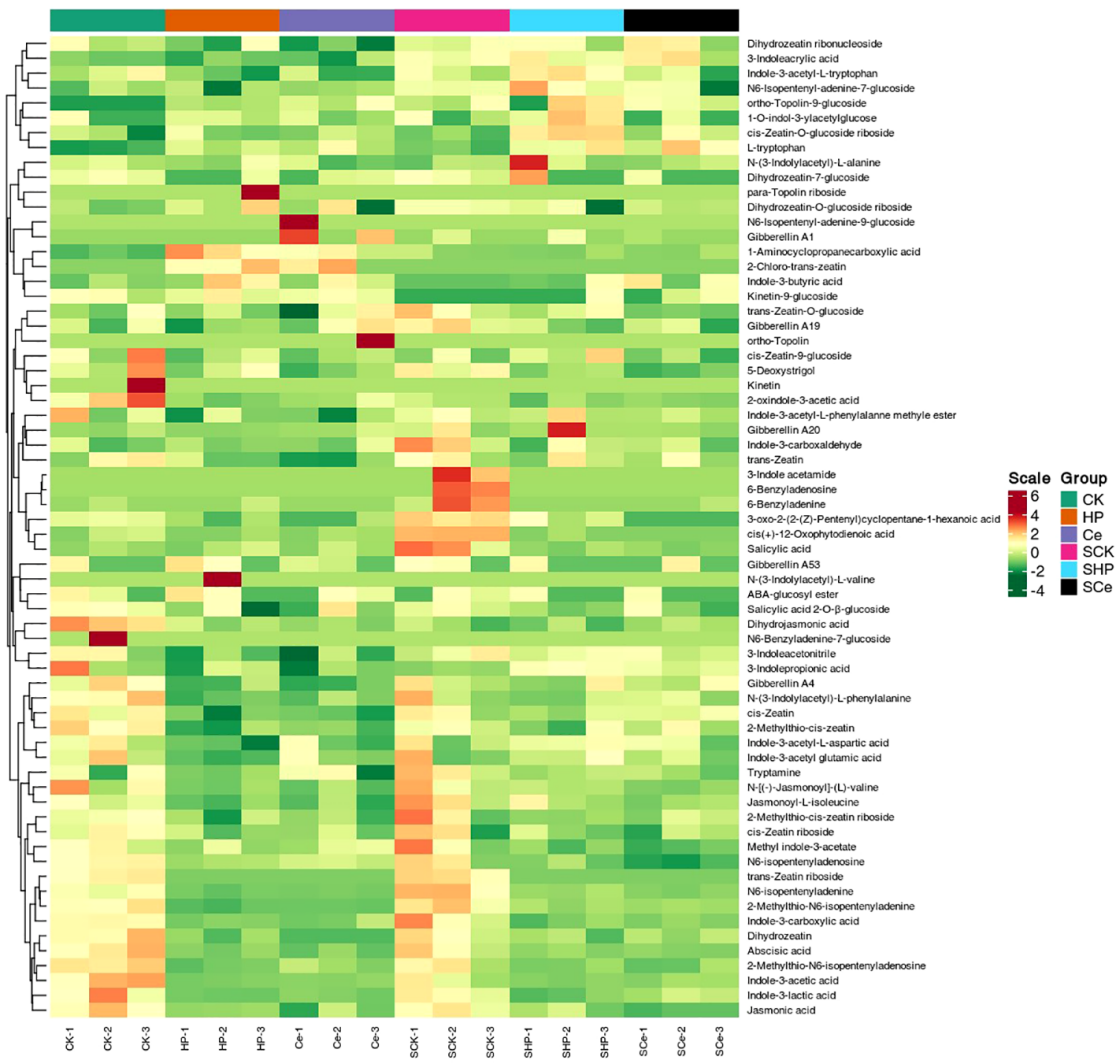
Heatmap of germinated seed metabolite levels under different treatments.

**Figure 8:**
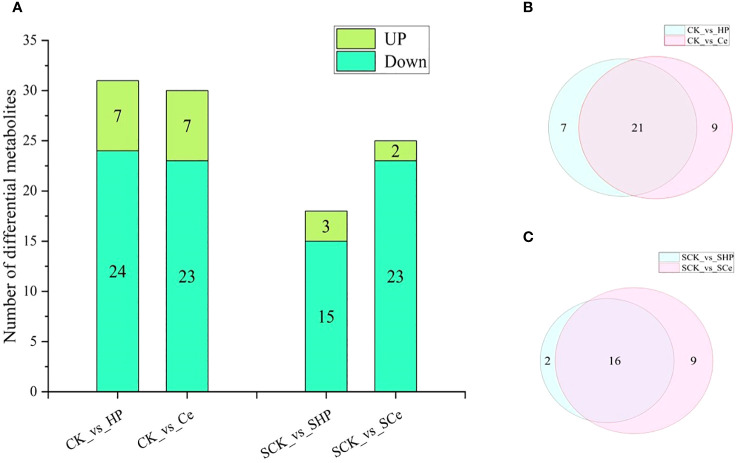
Statistical analysis of differential metabolites between different treatments. Differences in metabolite number between different treatments **(A)**. Venn diagram of control **(B)** and salt stress **(C)**.

As shown in **Figure 7**, hydropriming and CeO_2_NP priming downregulated the metabolites of cytokinins, auxins, and GAs compared with non-primed seeds. Under salt stress, hydropriming and CeO_2_NP priming downregulated JA, salicylic acid, GA, ABA, and strigolactones, as well as some metabolites related to auxins and cytokinins. The number of differential metabolites in the CK vs. HP comparison that were upregulated was 7 and that of metabolites that were downregulated was 24, which is 1 more downregulated metabolite compared with the CK vs. Ce comparison. The number of differential metabolites in the SCK vs. SHP comparison was 18, with 3 upregulated and 15 downregulated, which is 1 more upregulated and 8 fewer downregulated than in the SCK vs. SCe comparison (**Figure 8A**). CK vs. HP and CK vs. Ce comparisons had 7 and 9 unique differential metabolites, respectively, and 21 shared metabolites (**Figure 8B**). SCK vs. SHP and SCK vs. SCe comparisons had 2 and 9 unique differential metabolites, respectively, and 16 shared differential metabolites (**Figure 8C**).

### 3.11 Effects of different treatments on the metabolic pathways of alfalfa seeds

**Figure 9:**
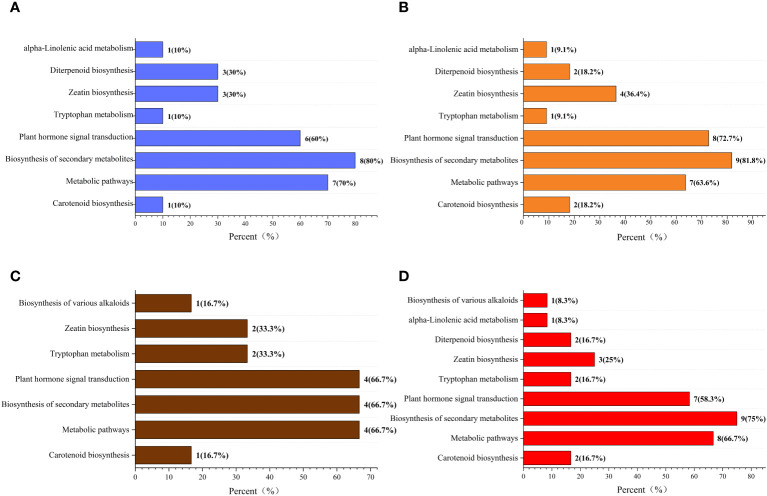
The Kyoto Encyclopedia of Genes and Genomes (KEGG) classification diagram of differential metabolites. Comparisons of CK vs. HP **(A)**, CK vs. Ce **(B)**, SCK vs. SHP **(C)**, and SCK vs. SCe **(D)**. The names of KEGG metabolic pathways are shown along the vertical axis, while the horizontal axis shows the number of differential metabolites annotated under each pathway and its corresponding proportion of the total number of metabolites annotated.

**Figure 10:**
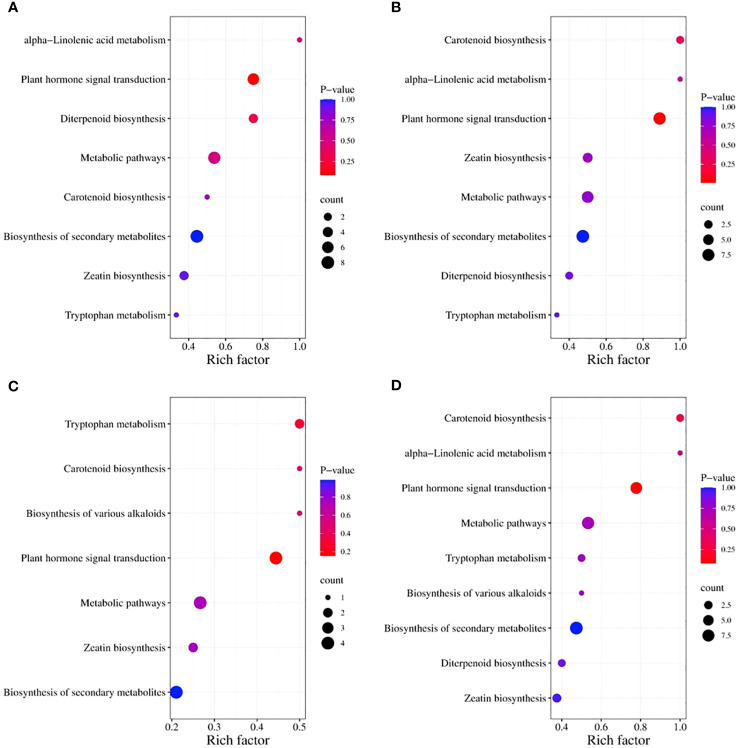
The enriched Kyoto Encyclopedia of Genes and Genomes (KEGG) pathways for differential metabolites in alfalfa seeds between different treatments. CK vs. HP **(A)**; CK vs. Ce **(B)**; SCK vs. SHP **(C)**; SCK vs. SCe **(D)**. The horizontal axis represents the rich factor corresponding to each pathway, the pathway names are shown along the vertical axis, and the color of each point corresponds to its *P*-value, i.e., the redder the point, the more significant the enrichment. The size of each point represents the number of enriched differential metabolites.

The Kyoto Encyclopedia of Genes and Genomes (KEGG) classification (**Figure 9**) and enrichment (**Figure 10**) analyses were conducted on the differential metabolites of alfalfa seeds germinated under CK, HP, and Ce treatments with and without salt stress. The KEGG pathway analysis between CK and HP indicates that significantly different metabolites were mainly enriched in α-linolenic acid metabolism, diterpenoid biosynthesis, zeatin biosynthesis, tryptophan metabolism, plant hormone signal transduction, biosynthesis of secondary metabolites, metabolic pathways, and carotenoid biosynthesis pathways. The numbers of significantly different metabolites in these metabolic pathways were one, three, three, one, six, eight, seven, and one, respectively (**Figure 9A**), and among these, the differences associated with the metabolism of α-linolenic acid and the enrichment of plant hormone signal transduction were relatively significant (**Figure 10A**). The KEGG classification of differential metabolites between CK and Ce treatments in the same pathways identified in the CK vs. HP comparison involved one, two, four, one, eight, nine, seven, and two differential metabolic pathways (**Figure 9B**), and the enrichment ratio of differential metabolites in the carotenoid biosynthesis and plant hormone signal transduction pathways was significant (**Figure 10B**). The differential metabolites between HP and Ce treatments were mainly enriched in diterpenoid biosynthesis, zeatin biosynthesis, plant hormone signal transduction, biosynthesis of secondary metabolites, and carotenoid biosynthesis pathways. The KEGG analysis (**Figures 9C**, **10C**) between seeds receiving no priming and hydropriming (SCK vs. SHP) under salt stress showed that there was a significant enrichment of one differential metabolite in the biosynthesis and carotenoid biosynthesis pathways of various alkaloids, with two differential metabolites enriched in the zeatin biosynthesis and tryptophan metabolism pathways and four differential metabolites among the plant hormone signal transduction, biosynthesis of secondary metabolites, and metabolic pathways. The KEGG analysis (**Figures 9D**, **10D**) of seeds under Ce treatment compared with non-priming under salt stress (SCK vs. SCe) showed that differential metabolites were mainly significantly enriched in the biosynthesis of various alkaloids, α-linolenic acid metabolism, diterpenoid biosynthesis, zeatin biosynthesis, tryptophan metabolism, plant hormone signal transduction, biosynthesis of secondary metabolites, metabolic pathways, and carotenoid biosynthesis pathways, with the number of significant differences in metabolites being one, one, two, three, two, seven, nine, eight, and two, respectively. The enrichment of carotenoid biosynthesis and plant hormone signal transduction pathways was significant. In addition, the KEGG analysis showed that compared with SHP, SCe induced α-linolenic acid metabolism and diterpenoid biosynthesis pathway enrichment of differential metabolites, and there were quantitative differences in the enrichment of differential metabolites in the zeatin biosynthesis, plant hormone signal transduction, biosynthesis of secondary metabolites, metabolic pathways, and carotenoid biosynthesis pathways.

## 4 Discussion

Our germination experiment results (**Figure 2**) indicated that the germination of alfalfa seeds and the growth of young seedlings are significantly affected by salt and that when the salt concentration reached 100 mM, the germination potential, germination rate, root length, and seedling length were significantly reduced, which is consistent with the observation that salt stress reduces plant seed germination rates and prolongs germination time (Munns, 2002). As the salt concentration increased, the germination rate and germination potential of alfalfa seeds decreased (**Figures 2A, B**), which is caused by multiple effects of salt stress. On the one hand, salinity changes the osmotic potential and inhibits the water absorption process of seeds (Munns and Tester, 2008), and on the other hand, excessive sodium and chloride ions have harmful effects on embryonic vitality (Daszkowska-Golec, 2011; Li et al., 2019). Based on calculations in the present study, the confirmed screening concentration (200 mM) is close to the results of Cui et al. (2022) for salt tolerance in ‘Huanghou’ alfalfa, the same alfalfa variety evaluated in the present study. We used CeO_2_NPs at a priming concentration of 0–500 mg/L for alfalfa seeds and found a significant increase in seed vigor under 200 mM of salt stress (**Figure 3**). Under such treatment conditions, the germination potential increased from 10.0% to 51.3%, the germination rate increased from 10.0% to 62.7%, and the root length increased from 8.3 cm to 23.1 cm (**Figure 3**), indicating a significant increase in priming effect and alleviation of salt stress. Similarly, previous studies have shown that CeO_2_NP priming can improve salt tolerance in the seeds of various species (Khan et al., 2021; Liu et al., 2021; Khan et al., 2022). For example, the germination rate of rapeseed increased by 12% under 200 mM of salt stress (Khan et al., 2021). As for the priming treatment, the phenomenon of seed germination and seedling growth first increasing and then decreasing with the increase of priming concentration (**Figures 3C, D**) is often considered to be associated with the toxicity of high-concentration nanomaterials to plants (Sun et al., 2023). In addition, the high osmotic potential of higher-concentration priming solutions may reduce water absorption and hinder germination.

Our results indicate that a priming concentration of 50 mg/L is optimal and superior to hydropriming (**Figure 3**). We can explain this from the perspectives of nanomaterial properties and seed coat structure (Khan et al., 2023). Firstly, the inherent characteristics of nanoparticles play an important role in the priming process. We found that the hydrodynamic size of CeO_2_NPs was >1,000 nm (**Figure 1C**), indicating that CeO_2_NPs showed agglomeration in aqueous solution (**Figure 1B**), which also appeared in the characterization results of Fe_2_O_3_ NPs (Sun et al., 2023). The size of nanomaterials directly affects the degree of the permeation potential and is related to whether they can penetrate the seed coat and enter the tissue to have an effect. Alfalfa, as a leguminous plant, has a typical hard seed, which shows that the impermeability of the seed coat hinders seed germination, and the wax layer on the seed coat surface plays an important role in this. The impermeability of the seed coat magnifies the osmotic stress caused by salt stress, leading to the slow initiation of the germination process. After priming, we found that the hard seed phenomenon was broken after CeO_2_NP priming. The gap between epidermal cells after CeO_2_NP priming was looser than that caused by hydropriming, and the wax layer was significantly cleared (**Figure 4C**). The gap generated in the epidermis could promote the water to enter more easily (Mahakham et al., 2016) and activate the α-amylase enzyme activity (Khan et al., 2023), which explained the observation of increased water absorption of CeO_2_NP-primed seeds (**Figure 4E**) and also may have provided a channel for the internal infiltration of CeO_2_NPs. Mahakham et al. (2017) found that NPs upregulated aquaporin regulator genes and promoted the increase of water absorption from a molecular perspective.

Additionally, we used EDX to analyze the distribution of elements in the epidermis, confirming that Ce was deposited on the seed coat (**Figure 4C**; **Table 3**). However, certain nanoparticles may also be beneficial for adsorption on the surface of the seed coating, and even if the nanoparticles are absorbed, most of them remain as a coating on the seed surface (Duran et al., 2017). In order to demonstrate the entry of CeO_2_NPs into the seed tissue, a line scan for Ce was conducted on seed sections, and it was found that Ce had indeed fully entered the seed and was evenly distributed in the tissue (**Figure 4D**). We conducted *in-vitro* experiments on CeO_2_NPs to assess their effect on α-amylase and found that CeO_2_NPs can improve the activity of α-amylase (6.55%) (**Figure 1E**), which is consistent with the determination of α-amylase activity (**Figure 5A**). Similarly, Ernest et al. (2012) previously found that AgNPs combined with α-amylase can be used for the rapid hydrolysis of starch, and a theoretical model consisting of an Ag–amylase complex (combination of nanomaterials and α-amylase) was proposed to improve enzyme catalytic efficiency. Therefore, it can be considered that the nanoenzyme characteristics of CeO_2_NPs have a direct effect on α-amylase activity, and there is an activation mechanism that needs to be explored urgently.

Amylase is the key hydrolase involved in seed germination, and it is related to the endosperm metabolism process in the germination stage and affects the seed germination speed. The present research found that the α-amylase activity of alfalfa seeds under CeO_2_NP priming was higher than that of non-primed and hydroprimed seeds, which may be the result of a combination of multiple factors, e.g., priming treatment accelerated water absorption (**Figure 4E**) and activated hydrolase; additionally, when CeO_2_NP enters cotyledons (**Figure 4D**) and has a nanocatalytic effect with intracellular α-amylase, it increases enzyme activity. Seed priming increased the activity of α-amylase and further increased the content of soluble sugar (**Figure 5B**). Nevertheless, this treatment did not increase α-amylase activity under salt stress, which may be due to the osmotic stress of the salt solution slowing down water absorption (Munns and Tester, 2008). Hydrolase can promote the production of soluble sugar, soluble protein, and free amino acid; provide nutrition for germination; and also act as an osmotic regulator to regulate osmotic potential and protect stressed cells.

In the present study, the relative conductivity and MDA content increased after priming treatment under control conditions (**Figures 6A, B**), and these are important indicators of the degree of membrane damage, indicating that the cell membrane was indeed damaged during the process of increasing water absorption and CeO_2_NP infiltration. ROS content was relatively reduced by nanopriming under salt stress (**Figures 6C, D**), and excessive ROS can lead to oxidative damage of membranes, proteins, and RNA and DNA molecules (Foyer et al., 2016). Therefore, priming treatments, especially nanopriming, reduced the relative conductivity and MDA content under salt stress (**Figures 6A, B**), indicating that CeO_2_NP priming treatment plays an active role in alleviating membrane damage of germinating alfalfa seeds under salt stress. In addition, CeO_2_NPs have excellent active ROS scavenging activity and have been used to protect plant photosynthesis from oxidative stress, especially under stress conditions (Wu et al., 2018). Most importantly, there are a large number of ROS detoxifying proteins in cells, such as CAT and SOD, which are able to alleviate oxidative stress (Foyer et al., 2016; Hasanuzzaman et al., 2020). In the present study, CeO_2_NPs induced an increase in CAT enzyme activity (**Figure 6G**); the corresponding increase in CAT activity removed excess H_2_O_2_ to protect cells from damage.

It has been reported that CeO_2_NPs can induce stress tolerance by regulating the level of plant hormones (Wang Y, et al., 2020). Among them, GA is the main hormone involved in seed germination and is responsible for activating hydrolase, while ABA has the opposite effect (Tong et al., 2017). Only when the ABA content decreases can GA play a role in promoting seed germination. JA and its derivatives inhibit seed germination (Dave et al., 2011; Liu et al., 2015), while ABA synergistically inhibits rice seed germination by promoting JA biosynthesis (Wang YF, et al., 2020). In the present study, CeO_2_NP priming induced a decrease in ABA and JA content compared with control seeds (**Figures 6H, I**). Therefore, reducing ABA and JA content is one of the key factors through which priming promoted seed germination and improved salt tolerance.

Compared with the control group, priming caused changes in multiple metabolic pathways, such as hormone signal transduction, which is the likely internal reason for significant changes in hormone content. However, the number of differential metabolites induced by CeO_2_NPs and hydropriming in the KEGG metabolic pathway was relatively similar. Notably, compared with hydropriming, CeO_2_NP priming promoted the enrichment of differential metabolites involved in carotenoid biosynthesis, plant hormone signal transduction, zeatin biosynthesis, and secondary metabolite biosynthesis pathways. Secondary metabolites play an important role in plant physiological adaptation to specific environmental conditions, resistance to biotic or abiotic stress, such as resistance to pests and diseases, and physiological adaptation to ecological changes (Dixon, 2001). Carotenoids can serve as signal molecule precursors for plant responses to external stimuli. In plants, carotenoids have effects such as promoting photomorphogenesis, participating in lipid peroxidation reactions (McNulty et al., 2007), and the biosynthesis of traditional plant hormones (such as ABA) and newly discovered plant hormones (such as unicorn lactone) (Umehara et al., 2008). Zeatin, a type of cytokinin, exhibited enrichment under CeO_2_NP priming, inducing accelerated growth of germinated seeds. Although the main priming factor affecting seed germination is water under non-stress conditions, CeO_2_NP priming still induced differential effects at the metabolic level. Under salt stress, compared with non-primed seeds, CeO_2_NP treatment induced a higher differential metabolic level than hydropriming. After the KEGG analysis, it was found that priming treatment affected hormone signal transduction, carotenoid synthesis, and other pathways to improve salt tolerance. The difference between CeO_2_NP priming and hydropriming was mainly concentrated in the main α-linolenic acid metabolism and diterpenoid biosynthesis pathways. α-Linolenic acid is an important component of cell membranes, and plants can respond to biotic and abiotic stresses by releasing α-linolenic acid, reshaping the fluidity of their membranes (Iba, 2002); additionally, free α-linolenic acid is a signaling molecule under stress. In short, from a metabolic perspective, seed priming appears to have promoted seed germination by regulating metabolic pathways such as those involved with carotenoids, zeatin, and plant hormone signal transduction under non-stress conditions. Under salt stress, CeO_2_NP priming enhanced seed salt tolerance by stimulating metabolic pathways such as those associated with α-linolenic acid and diterpenoids.

## 5 Conclusion

We used CeO_2_NPs to prime alfalfa seeds to promote seed germination under salt stress and explored aspects such as seed coat structure, the protective enzyme system, and key hormone metabolism levels. Before the germination experiment, we characterized NPs using methods that included TEM and XRD. The results showed that 1) CeO_2_NP priming at a concentration of 0–500 mg/L could significantly alleviate 200 mM of salt stress in alfalfa seeds, with 50 mg/L having the best alleviating effect; 2) compared with hydropriming, CeO_2_NP priming had a better effect and induced a more significant improvement in salt tolerance of alfalfa seeds; and 3) CeO_2_NPs have nanoenzyme catalytic activity, which was enhanced in the *in-vitro* experiments evaluating α-amylase activity. By reducing the hardness of seeds, priming increased seed water absorption and promoted the internalization of Ce elements into the seeds while reducing ABA and JA contents, thus relieving seed dormancy. CeO_2_NP priming increased α-amylase activity and the levels of osmoregulatory substances to promote seed germination and alleviate osmotic stress caused by salinity. By reducing ROS, MDA content, and relative conductivity and increasing CAT enzyme activity, oxidative damage caused by salt stress was alleviated. Seed priming promoted seed germination by regulating metabolic pathways such as those involved with carotenoid, zeatin, and plant hormone signal transduction under non-stress conditions, and CeO_2_NP priming was able to enhance seed salt tolerance by regulating the metabolic pathways associated with α-linolenic acid and diterpenoid hormones.

In summary, CeO_2_NPs can effectively enhance seed vigor under non-stress conditions and significantly improve the salt tolerance of seeds. A simple seed treatment strategy that can enhance crop stress resistance is thus proposed, i.e., treatment of seeds with CeO_2_NPs, which is of great importance for improving crop stress resistance, reducing farmland investment, and promoting sustainable agricultural development.

## Data availability statement

The original contributions presented in the study are included in the article/supplementary material. Further inquiries can be directed to the corresponding author.

## Author contributions

JG: Conceptualization, Data curation, Formal analysis, Investigation, Methodology, Writing – original draft. YL: Data curation, Investigation, Writing – review & editing. DZ: Writing – review & editing. YD: Investigation, Writing – review & editing. LG: Data curation, Investigation, Writing – review & editing. XS: Data curation, Investigation, Writing – review & editing. KS: Conceptualization, Methodology, Writing – review & editing. XH: Conceptualization, Formal analysis, Funding acquisition, Methodology, Resources, Supervision, Writing – review & editing.

## References

[B1] Aebi H. (1984). Catalase *in vitro* . Method Enzymol. 105, 121–126. doi: 10.1016/S0076-6879(84)05016-3 6727660

[B2] Amritha M. S. Sridharan K. Puthur J. T. Dhankher O. P. (2021). Priming with nanoscale materials for boosting abiotic stress tolerance in crop plants. J. Agric. Food Chem. 69 (35), 10017–10035. doi: 10.1021/acs.jafc.1c03673 34459588

[B3] Barradas J. M. M. Abdelfattah A. Matula S. Dolezal F. (2015). Effect of fertigation on soil salinization and aggregate stability. J. Irrigation Drainage Eng. 141 (4), 1–7. doi: 10.1061/(ASCE)IR.1943-4774.0000806

[B4] Beauchamp C. Fridovich I. (1971). Superoxide dismutase: improved assays and an assay applicable to acrylamide gels. Anal. Biochem. 44, 276–287. doi: 10.1016/0003-2697(71)90370-8 4943714

[B5] Bradford M. M. (1976). A rapid and sensitive method for the quantitation of microgram quantities of protein utilizing the principle of protein-dye binding. Analytical Biochem. 72, 248–254. doi: 10.1016/0003-2697(76)90527-3 942051

[B6] Chen H. Zhao G. Li Y. Wang D. Ma Y. (2019). Monitoring the seasonal dynamics of soil salinization in the Yellow River delta of China using Landsat data. Natural Hazards Earth System Sci. 19 (7), 1499–1508. doi: 10.5194/nhess-19-1499-2019

[B7] Cui X. W. Guo Z. P. Mao Y. Niu J. P. Sui X. Xu N.. (2022). Evaluation on salt tolerance of 33 alfalfa cultivars during germination. J. Domest. Anim. Ecol. 43 (02), 55–65. doi: 10.3969/ji.ssn.1673-1182.2022.02.009

[B8] Damaris R. N. Lin Z. Y. Yang P. F. He D. L. (2019). The rice alpha-amylase, conserved regulator of seed maturation and germination. Int. J. Mol. Sci. 20 (2), 450. doi: 10.3390/ijms20020450 30669630 PMC6359163

[B9] Daszkowska-Golec A. (2011). Arabidopsis seed germination under abiotic stress as a concert of action of phytohormones. OMICS: A J. Integr. Biol. 15 (11), 763–774. doi: 10.1089/omi.2011.0082 22011341

[B10] Dave A. Hernandez M. L. He Z. S. Andriotis V. M. E. Vaistij F. E. Larson T. R.. (2011). 12-oxo-phytodienoic acid accumulation during seed development represses seed germination in Arabidopsis. Plant Cell 23 (2), 583–599. doi: 10.1105/tpc.110.081489 21335376 PMC3077774

[B11] Dixon R. A. (2001). Natural products and plant disease resistance. Nature 411 (6839), 843–847. doi: 10.1038/35081178 11459067

[B12] Do E. S. P. A. Caixeta O. H. Fernandes F. L. Santaella C. (2021). Nanotechnology potential in seed priming for sustainable agriculture. Nanomaterials 11 (2), 267. doi: 10.3390/nano11020267 33498531 PMC7909549

[B13] Duran N. M. Savassa S. M. de Lima R. G. de Almeida E. Linhares F. S. van Gestel C.. (2017). X-ray spectroscopy uncovering the effects of Cu based nanoparticle concentration and structure on phaseolus vulgaris germination and seedling development. J. Agric. Food Chem. 65 (36), 7874–7884. doi: 10.1021/acs.jafc.7b03014 28817280

[B14] El-Hendawy S. Elshafei A. Al-Suhaibani N. Alotabi M. Hassan W. Dewir Y. H.. (2019). Assessment of the salt tolerance of wheat genotypes during the germination stage based on germination ability parameters and associated SSR markers. J. Plant Interact. 14 (1), 151–163. doi: 10.1080/17429145.2019.1603406

[B15] Ferguson I. B. Watkins C. B. Harman J. E. (1983). Inhibition by calcium of senescence of detached cucumber cotyledons: effect on ethylene and hydroperoxide production. Plant Physiol. 71 (1), 182–186. doi: 10.1104/pp.71.1.182 16662782 PMC1066009

[B16] Foyer C. H. Rasool B. Davey J. W. Hancock R. D. (2016). Cross-tolerance to biotic and abiotic stresses in plants: a focus on resistance to aphid infestation. J. Exp. Bot. 67 (7), 2025–2037. doi: 10.1093/jxb/erw079 26936830

[B17] Guillaume N. Yong X. Wim J. J. S. (2017). The release of dormancy, a wake-up call for seeds to germinate. Curr. Opin. Plant Biol. 35, 8–14. doi: 10.1016/j.pbi.2016.09.002 27710774

[B18] Hanin M. Ebel C. Ngom M. Laplaze L. Masmoudi K. (2016). New insights on plant salt tolerance mechanisms and their potential use for breeding. Front. Plant Sci. 7. doi: 10.3389/fpls.2016.01787 PMC5126725 27965692

[B19] Hasanuzzaman M. Bhuyan M. Zulfiqar F. Raza A. Mohsin S. M. Al Mahmud J.. (2020). Reactive oxygen species and antioxidant defense in plants under abiotic stress: revisiting the crucial role of a universal defense regulator. Antioxidants 9 (8), 681. doi: 10.3390/antiox9080681 32751256 PMC7465626

[B20] Heath R. L. Packer L. (1968). Photoperoxidation in isolated chloroplasts: I. Kinetics stoichiometry Fatty Acid peroxidation. Arch. Biochem. Biophys. 125, 189–198. doi: 10.1016/0003-9861(68)90654-1 5655425

[B21] Hemeda H. M. Klein B. P. (1990). Efects of naturally occurring antioxidants on peroxidase activity of vegetable extracts. J. Food Sci. 55, 184–185. doi: 10.1111/j1365-2621.1990.tb06048.x

[B22] Iba K. (2002). Acclimative response to temperature stress in higher plants: approaches of gene engineering for temperature tolerance. Annu. Rev. Plant Biol. 53, 225–245. doi: 10.1146/annurev.arplant.53.100201.160729 12221974

[B23] Ibrahim E. A. (2016). Seed priming to alleviate salinity stress in germinating seeds. J. Plant Physiol. 192, 38–46. doi: 10.1016/j.jplph.2015.12.011 26812088

[B24] Kaneko M. Itoh H. Ueguchi-Tanaka M. Ashikari M. Matsuoka M. (2002). The alpha-amylase induction in endosperm during rice seed germination is caused by gibberellin synthesized in epithelium. Plant Physiol. 128 (4), 1264–1270. doi: 10.1104/pp.010785 11950975 PMC154254

[B25] Ke D. S. Wang A. G. Sun G. C. Dong L. F. (2002). The effect of active oxygen on the activity of ACC synthase induced by exogenous IAA. Acta Botanica Sin. 44 (5), 551–556.

[B26] Khan M. N. Fu C. Li J. Tao Y. Li Y. Hu J.. (2023). Seed nanopriming: How do nanomaterials improve seed tolerance to salinity and drought? Chemosphere 310, 136911. doi: 10.1016/j.chemosphere.2022.136911 36270526

[B27] Khan M. N. Li Y. H. Fu C. C. Hu J. Chen L. L. Yan J. S.. (2022). CeO_2_ nanoparticles seed priming increases salicylic acid level and ros scavenging ability to improve rapeseed salt tolerance. Global Challenges 6 (7), 1–13. doi: 10.1002/gch2.202200025 PMC9284644 35860396

[B28] Khan M. N. Li Y. Khan Z. Chen L. Liu J. Hu J.. (2021). Nanoceria seed priming enhanced salt tolerance in rapeseed through modulating ROS homeostasis and alpha-amylase activities. J. Nanobiotechnology 19 (1), 276. doi: 10.1186/s12951-021-01026-9 34530815 PMC8444428

[B29] Kishorekumar A. Jaleel C. A. Manivannan P. Sankar B. Sridharan R. Panneerselvam R. (2007). Comparative effects of different triazole compounds on growth, photosynthetic pigments and carbohydrate metabolism of Solenostemon rotundifolius. Colloids Surf B Biointerfaces 60 (2), 207–212. doi: 10.1016/j.colsurfb.2007.06.008 17669636

[B30] Lee S. C. Luan S. (2012). ABA signal transduction at the crossroad of biotic and abiotic stress responses. Plant Cell Environ. 35 (1), 53–60. doi: 10.1111/j.1365-3040.2011.02426.x 21923759

[B31] Li M. Y. Wang Y. Liang D. N. Yao Y. N. Lan J. (2019). Comprehensive evaluation of salt tolerance of 22 alfalfa germplasms at germination stage. Acta Agriculturae Zhejiangensis 31 (05), 746–755. doi: 10.3969/j.issn.1004-1524.2019.05.10

[B32] Liu J. Li G. Chen L. Gu J. Wu H. Li Z. (2021). Cerium oxide nanoparticles improve cotton salt tolerance by enabling better ability to maintain cytosolic K^+^/Na^+^ ratio. J. Nanobiotechnology 19 (1), 153. doi: 10.1186/s12951-021-00892-7 34034767 PMC8146236

[B33] Liu Z. Zhang S. M. Sun N. Liu H. Y. Zhao Y. H. Liang Y. L.. (2015). Functional diversity of jasmonates in rice. Rice 8 (1), 1–13. doi: 10.1186/s12284-015-0042-9 26054241 PMC4773313

[B34] Mahakham W. Sarmah A. K. Maensiri S. Theerakulpisut P. (2017). Nanopriming technology for enhancing germination and starch metabolism of aged rice seeds using phytosynthesized silver nanoparticles. Sci. Rep. 7, 1–21. doi: 10.1038/s41598-017-08669-5 28811584 PMC5557806

[B35] Mahakham W. Theerakulpisut P. Maensiri S. Phumying S. Sarmah A. K. (2016). Environmentally benign synthesis of phytochemicals-capped gold nanoparticles as nanopriming agent for promoting maize seed germination. Sci. Total Environ. 573, 1089–1102. doi: 10.1016/j.scitotenv.2016.08.120 27639594

[B36] Marthandan V. Geetha R. Kumutha K. Renganathan V. G. Karthikeyan A. Ramalingam J. (2020). Seed priming: a feasible strategy to enhance drought tolerance in crop plants. Int. J. Mol. Sci. 21 (21), 8258. doi: 10.3390/ijms21218258 33158156 PMC7662356

[B37] McNulty H. P. Byun J. Lockwood S. F. Jacob R. F. Mason R. P. (2007). Differential effects of carotenoids on lipid peroxidation due to membrane interactions: X-ray diffraction analysis. Biochim. Biophys. Acta 1768 (1), 167–174. doi: 10.1016/j.bbamem.2006.09.010 17070769

[B38] Migaszewski Z. M. Galuszka A. (2015). The characteristics, occurrence, and geochemical behavior of rare earth elements in the environment: a review. Crit. Rev. Environ. Sci. Technol. 45 (5), 429–471. doi: 10.1080/10643389.2013.866622

[B39] Miller G. Suzuki N. Ciftci-Yilmaz S. Mittler R. (2010). Reactive oxygen species homeostasis and signalling during drought and salinity stresses. Plant Cell Environ. 33 (4), 453–467. doi: 10.1111/j.1365-3040.2009.02041.x 19712065

[B40] Munns R. (2002). Comparative physiology of salt and water stress. Plant Cell Environ. 25 (2), 239–250. doi: 10.1046/j.0016-8025.2001.00808.x 11841667

[B41] Munns R. Tester M. (2008). Mechanisms of salinity tolerance. Annu. Rev. Plant Biol. 59, 651–681. doi: 10.1146/annurev.arplant.59.032607.092911 18444910

[B42] Pourkhorsandi H. Debaille V. de Jong J. Armytage R. (2021). Cerium stable isotope analysis of synthetic and terrestrial rock reference materials by MC-ICPMS. TALANTA 224, 121877. doi: 10.1016/j.talanta.2020.121877 33379086

[B43] Qin H. Huang R. F. (2020). The phytohormonal regulation of Na^+^/K^+^ and reactive oxygen species homeostasis in rice salt response. Mol. Breed. 40 (5), 1–13. doi: 10.1007/s11032-020-1100-6

[B44] Rajendran K. Tester M. Roy S. J. (2009). Quantifying the three main components of salinity tolerance in cereals. Plant Cell Environ. 32 (3), 237–249. doi: 10.1111/j.1365-3040.2008.01916.x 19054352

[B45] Rumanti I. A. Hairmansis A. Nugraha Y. Nafisah Susanto U. Wardana P. Subandiono R. E.. (2018). Development of tolerant rice varieties for stress-prone ecosystems in the coastal deltas of Indonesia. Field Crops Res. 223, 75–82. doi: 10.1016/j.fcr.2018.04.006

[B46] Song K. X. Gao J. Z. Li S. Sun Y. F. Sun H. Y. An B. Y.. (2021). Experimental and theoretical study of the effects of rare earth elements on growth and chlorophyll of alfalfa (*Medicago sativa* L.) seedling. Front. Plant Sci. 12, 731838. doi: 10.3389/fpls.2021.731838 34691110 PMC8531810

[B47] Sun H. Y. Qu G. P. Li S. Song K. X. Zhao D. H. Li X.. (2023). Iron nanoparticles induced the growth and physio-chemical changes in *Kobresia capillifolia* seedlings. Plant Physiol. Biochem. 194, 15–28. doi: 10.1016/j.plaphy.2022.11.001 36368222

[B48] Tanou G. Fotopoulos V. Molassiotis A. (2012). Priming against environmental challenges and proteomics in plants: update and agricultural perspectives. Front. Plant Sci. 3. doi: 10.3389/fpls.2012.00216 PMC3438484 22973291

[B49] Tong S. M. Xi H. X. Ai K. J. Hou H. S. (2017). Overexpression of wheat TaNCED gene in Arabidopsis enhances tolerance to drought stress and delays seed germination. Biol. plantarum 61, 64–72. doi: 10.1007/s10535-016-0692-5

[B50] Umehara M. Hanada A. Yoshida S. Akiyama K. Arite T. Takeda-Kamiya N.. (2008). Inhibition of shoot branching by new terpenoid plant hormones. Nature 455 (7210), 195–200. doi: 10.1038/nature07272 18690207

[B51] Ernest E. Shiny P. J. Mukherjee A Chandrasekaran N (2012). Silver nanoparticles: a potential nanocatalyst for the rapid degradation of starch hydrolysis by a-amylase. Carbohydr. Res. 352, 60–64. doi: 10.1016/j.carres.2012.02.009 22405762

[B52] Wang Y. F. Hou Y. X. Qiu J. H. Wang H. M. Wang S. Tang L. Q.. (2020). Abscisic acid promotes jasmonic acid biosynthesis via a ‘SAPK10-b ZIP72-AOC’pathway to synergistically inhibit seed germination in rice (*Oryza sativa*). New Phytol. 228 (4), 1336–1353. doi: 10.1111/nph.16774 32583457 PMC7689938

[B53] Wang Y. Y. Wang L. Q. Ma C. X. Wang K. X. Hao Y. Chen Q.. (2019). Effects of cerium oxide on rice seedlings as affected by co-exposure of cadmium and salt. Environ. pollut. 252, 1087–1096. doi: 10.1016/j.envpol.2019.06.007 31252106

[B54] Wang Y. Zhang P. Li M. Guo Z. Ullah S. Rui Y.. (2020). Alleviation of nitrogen stress in rice (*Oryza sativa*) by ceria nanoparticles. Environ. Science: Nano 7, 2930–2940. doi: 10.1039/D0EN00757A

[B55] Wu H. H. Shabala L. Shabala S. Giraldo J. P. (2018). Hydroxyl radical scavenging by cerium oxide nanoparticles improves Arabidopsis salinity tolerance by enhancing leaf mesophyll potassium retention. Environ. Science-Nano 5 (7), 1567–1583. doi: 10.1039/C8EN00323H

[B56] Yang Y. Guo Y. (2018). Elucidating the molecular mechanisms mediating plant salt-stress responses. New Phytol. 217 (2), 523–539. doi: 10.1111/nph.14920 29205383

[B57] Yang J. X. Zhao J. Zhu G. F. Wen Y. Y. Wang Y. Q. Liu J. L.. (2022). Effects of ecological water conveyance on soil salinization in the Shiyang River Basin's terminal Lake-Qingtu Lake-Area. Sustainability 14 (10311), 1–11. doi: 10.3390/su141610311

[B58] Zhao L. J. Bai T. H. Wei H. Gardea-Torresdey J. L. Keller A. White J. C. (2022). Nanobiotechnology-based strategies for enhanced crop stress resilience. Nat. Food 3 (10), 829–836. doi: 10.1038/s43016-022-00596-7 37117882

[B59] Zou Q. (2003). Experimental guidance of plant physiology (Beijing: China Agriculture Press).

